# Generalization of the Receiver-Operating Characteristic Curve to Determine the Normal Hemoglobin Range Cutoff Points in Pregnant Women

**DOI:** 10.5812/ircmj.18318

**Published:** 2014-07-05

**Authors:** Hamid Alavi Majd, Ahmad Reza Baghestani, Nasrin Broumandnia, Nourossadat Kariman, Nastaran Safavi Ardebili, Elham Sajjadi

**Affiliations:** 1 Department of Biostatistics, School of Paramedical Sciences, Shahid Beheshti University of Medical Sciences, Tehran, IR Iran; 2 Research Institute for Reproductive Health, Faculty of Nursing and Midwifery, Shahid Beheshti University of Medical Sciences, Tehran, IR Iran; 3 International Branch of Shahid Beheshti University of Medical Sciences, Tehran, IR Iran; 4 Department of Hematology, Tarbiat Modares University, Tehran, IR Iran

**Keywords:** ROC curve, Sensitivity and Specificity, Biomarkers, Pregnancy Outcomes

## Abstract

**Background:**

Identification of a normal range for biomarkers, based on pregnancy outcomes (caused by their high or low values) is of special importance in clinical studies. As some pregnancy outcomes can happen in both high and low levels of biomarkers, the receiver-operating characteristic (ROC) curve is unsuitable for identifying these levels separately; rather, a statistical method is preferable which identifies both levels simultaneously.

**Objectives:**

To this effect, our research introduces a generalization of ROC curve (by using a number of related consequences) to identify a normal range for the biomarker. Practically, the study intends to identify a normal range of hemoglobin in the first trimester of pregnancy to prevent adverse outcomes that can be caused by high and low levels of hemoglobin.

**Patients and Methods:**

The current article introduces an ROC generalization curve to determine a normal range for biomarkers based on a number of pregnancy outcomes, which may occur in high and low levels of biomarkers. Simulated data were also used to compare the current method with the ROC curve method. Our data collected from a cohort study carried out on 600 pregnant women referring to Milad Hospital in Tehran, Iran in 2010. The data comprised hemoglobin level in the first trimester of pregnancy as well as pregnancy outcomes such as preterm delivery, low birth weight, preeclampsia, and gestational diabetes. We calculated an estimation of the normal range of hemoglobin for the study population. Statistical analysis was carried out by R software, version 3.0.2.

**Results:**

Results from the simulation study indicated that, the new method was better than the methods which used two ROC curves separately with regard to sensitivity and specificity. In this method, the level of normal hemoglobin in the first trimester ranged from 10 to 12.4 with sensitivity and specificity levels of 76.2% and 48% respectively, which is higher than previous studies.

**Conclusions:**

With regard to the normal range of biomarkers, our method yielded greater sensitivity and specificity levels than methods using the ROC curve, which separately analyzes the data, particularly in occasions with common consequences in high and low levels of the biomarker.

## 1. Background

The ROC (Receiver Operating Characteristic) curve is widely used to identify cutoff points in biomarkers (1). Sometimes, a rising or a declining pattern in a biomarker could indicate the emergence of a more serious phase of a disease. In such occasion, the generalized Youden’s index and the ROC surface could be used to select cutoff points (2-4).

Nonetheless, other types of questions arise in clinical issues, as they mostly deal with identification of a normal range for biomarkers, and certain pregnancy outcomes can happen at both the high and low levels of biomarkers.

The statistical method used in most clinical researches consists of two separate ROC curves to detect cutoff points for the normal range; in other words, one ROC curve to detect a cutoff point for the high level of the normal range and another one for the lower level. However, in some other studies, clinical experiences are merely used to detect an approximate range for certain biomarkers. The application of such methods, along with common unpleasant results, will cause some problems that are discussed below.

As indicated earlier, in certain situations, unpleasant outcomes happen at very low and very high levels of biomarkers. For example, both high and low levels of hemoglobin during pregnancy can cause low birth weight (5). Noticeably, in such cases, if two separate ROC curves are used to detect the low and high level cutoff points of the biomarker, the shared aspects of such consequences would be ignored. To clarify the issue, suppose that both the high and low levels of biomarker "A" are warning signs for contracting disease "D". We aim to identify a normal range for the biomarker "A", so that the possibility of the contracting disease “D” would considerably decrease if the individual had a biomarker at this range.

Now, suppose a researcher uses an ROC curve to detect an upper cutoff point with the assumption that those with biomarker level above this point are likely to catch disease "D", while those with a biomarker below this point would remain healthy. Then, some individuals who develop disease "D" due to the low level of biomarker "A" have been mistakenly considered healthy, which would affect sensitivity and specificity levels. Likewise, if another ROC curve were used to detect a lower cutoff point (without considering the fact that the patients with high levels of biomarkers can contract the disease), the same problem would arise.

Hence, the application of two ROC curves which separately identify normal ranges for the biomarker is inappropriate in these circumstances. Instead, simultaneous selection of two cutoff points of the normal range for the biomarker is necessary in order to foresee the shared unpleasant outcomes at both (high and low) levels of biomarkers; this approach could help to identify the best normal range of a biomarker with the highest sensitivity and specificity.

## 2. Objectives

In the present article, a statistical method, derived from generalization of the ROC curve, is introduced for simultaneous identification of two cutoff points and detection of a biomarker normal range. Unlike the ROC curve, which considers just one disease, the current method could take into account several diseases and their interrelated consequences due to high or low levels of the biomarker. Another considerable and distinctive feature of the method is considering shared outcomes at both high and low levels of biomarker.

As for the practical application, the article uses the relationship between hemoglobin levels of pregnant women during the first trimester of pregnancy, with pregnancy outcomes, and then identifies the normal range of hemoglobin.

The low level of hemoglobin in pregnancy could cause adverse pregnancy outcomes such as intrauterine growth disorder, or death, preterm delivery, and low birth weight (6). The relation between hemoglobin level and lower birth weight (7, 8), and preterm delivery (9) tends to form a u-shaped curve. In other words, both low and high levels of hemoglobin are risk factors for low birth weight and preterm delivery (10, 11). In different studies, an increase in hemoglobin density during the first half of pregnancy is shown to be a risk factor for contracting preeclampsia (12, 13) and diabetes (14). Considering the above unpleasant results and the fact that some of the consequences are shared, the statistical method presented in this research is used to detect the normal range of hemoglobin during the first trimester of pregnancy.

## 3. Patients and Methods

Considering the importance of the subject, this section introduces a statistical method to identify a normal range of biomarker based on a number of diseases caused by high or low levels of biomarker. The method will also include unpleasant results that can happen due to high or low levels of the biomarker.

### 3.1. Statistical Method

The suggested statistical method can be successfully applied according to the algorithm which follows. It is to be noted that the rationale underlying this method is similar to that of the ROC curve analysis.

The first phase: Initially, the first cutoff point (the lower level of the normal range) is considered by using the lowest point of the biomarker. Then, the second cutoff point (the upper level of normal range) will be set one step after the first cutoff point in order that the highest level of biomarker is changed (the intended step is determined based on the significance of the biomarker domain).

C_1_ = min (biomarker)

C_2_ = C_1_ + step, C_1_ + 2 step, C_1_ + 3 step, ...

Until C_2_ < max (biomarker)

The second phase: The below contingency table (Table 1) is necessary to determine sensitivity and specificity, based on each cutoff point pairs in previous phases.

**Table 1.** Contingency Table

The Identified Condition	Biomarker Less Than the First Cutoff Point	Biomarker Between Two Cutoff Points	Biomarker More Than the Second Cutoff Point
**The true condition**			
Having at least one of the consequences due to low level of biomarker	n _1_	n _2_	n _3_
Healthy	n _4_	n _5_	n _6_
With at least one of the consequences due to high level of biomarker	n _7_	n _8_	n _9_

$$\mathrm{Specificity}=\frac{n5}{n4+n5+n6}$$

$$\mathrm{Sensivity}=\frac{n1+n9}{n1+n2+n3+n7+n8+n9}$$

In the current article, only sensitivity and specificity have been computed; however, other evaluation indexes like accuracy, positive predictive value and negative predictive value could also be computed in the same way.

The first cutoff point is moved to the next step, and the later process is repeated.

C_1_ = C_1_ (before) + step

Do until C_1_ < max (biomarker)

It is worth mentioning that, as long as the first cutoff point is less than the highest level in the biomarker domain, the process will continue. In this stage, whenever the algorithm is stopped (by evaluating the identified sensitivity and specificity), the normal range with the highest sensitivity and specificity levels has to be chosen.

C_1_ , C_2_ = max_C1, C2_ (Sensitivity, Specificity)

The method presented in this article was administered by pROC package and also by developing a program in the "R" software, the 3.0.2 version, and its code is available if the reader contact with the corresponding author.

### 3.2. Simulation

To compare the results of the suggested method with those of the ROC curve, a simulation study was carried out. The technique of administering the simulated method was as follows. First, a biomarker with a normal distribution (mean = 12 and standard deviation = 1), in four sample sizes of 50, 100, 200, and 500 was generated. As biomarkers usually follow a normal distribution in the society, we chose a normal distribution. The selected mean and standard deviation were the current study suggestions and, accordingly, the administration of similar studies with any other values for the mean and standard deviation would also be possible.

The normal range of biomarker was considered as follows: (12.6 ± 1) µ ± σ. Then, using a binomial distribution, with P = 0.9, 90% of samples, whose biomarkers went beyond the normal range of < 11.6 or > 13.6 were considered as patients. Thus, common consequences at high and low levels of the biomarker were also considered. In the next step, the ROC curve method and the current study method were used to analyze the data and in this way, the sensitivity and specificity values, for both methods, were computed. It is to be noted that, for each sample size, the simulated steps were repeated 100 times and, in the end, the averages for sensitivity and specificity were reported. Table 2 presents the simulation results.

**Table 2.** Results from the Application of the Current Study Method and ROC Curve for the Simulated Biomarker

Sample Size	The Suggested Method		ROC Curve Method			
			if C = 13.6		if C = 11.6	
	Specificity	Sensitivity	Specificity	Sensitivity	Specificity	Sensitivity
**n = 50**	93.55	77.25	65.5	80.85	44	61.5
**n = 100**	93.55	85.15	64.5	51	38.6	59.45
**n = 200**	94.65	81.3	22.5	57.6	79.65	48.25
**n = 500**	93.85	79.3	49.75	54.05	50.05	51.2
**n = 600**	93.55	79.4	33.3	58.15	69.1	48.75

For example, a careful look at results from the sample size 100, would indicate that the suggested method yields sensitivity and specificity of 85.15 and 93.55, respectively. However, if two ROC curves are used to compute two separate values for upper and lower levels, then considering the lower cutoff point (i.e. 11.6), the sensitivity and specificity will be 59.45 and 38.6, respectively. Furthermore, considering the upper level of the cutoff point (i.e. 13.6), the sensitivity and specificity will be 51 and 64.5, respectively; such figures clearly show that the suggested method produces much better results than the methods computing the high and low levels separately. Similar results were observed in other sample sizes. To achieve a desired sensitivity and specificity level with the ROC curve, the researcher has to look for some other cutoff points, which are not the true normal values; this is because, when the ROC curve is used, neither sensitivity nor specificity could be suitable options for a true cutoff point.

### 3.3. Data of the study

The data used in this cohort study included the hemoglobin level of 600 pregnant women, during their first trimester, who referred to Milad Hospital in Tehran, Iran in 2010. The level of the hemoglobin during their first trimester of pregnancy was recorded at Milad Hospital. The participants were then followed until delivery and the pregnancy outcomes, including preterm delivery, low birth weight, preeclampsia and gestational diabetes were recorded. The characteristics of our data such as inclusion and exclusion criteria, measurement instruments, validity and reliability of measurements and the methods of assessment of the data have been described in study of Safavi Arbedili et al. in detail (13).

To administer the suggested method, the participants with at least one of these unpleasant outcomes were considered "unhealthy" and the rest of them were considered "healthy". In other words, data from several diseases were simultaneously used to detect a normal range for the hemoglobin biomarker.

## 4. Results

Out of 600 pregnant women under study, 172 (28.7%) women had been afflicted with at least one of the discomforts of preterm delivery, low birth weight, preeclampsia, or diabetes while the remaining 428 (71.3%) women were shown to be healthy. Moreover, in the current sample, there were no cases of stillbirth or intrauterine growth problem. Using the present method, the best cutoff points (normal range) for the hemoglobin during the first trimester were identified as 10 and 12.4 with sensitivity and specificity of 76.2% and 48%, respectively. Therefore, with this normal range of hemoglobin, we can accurately predict the health status of 76.2% of mothers who will contract one of the mentioned adverse pregnancy outcomes. Also, some other cutoff points with sensitivity and specificity similar to the above normal levels appear in Table 3. Thus, keeping in mind the significance of sensitivity and specificity, a clinician can choose the ideal range.

**Table 3.** The Sensitivity (%) and Specificity (%) for Suggested Normal Range

Recommended Normal Range for Hemoglobin (Lower Limits, Upper Limits)	Sensitivity	Specificity
**9.1-12.4**	75.6	47.7
**9.8-12.4**	76.2	47.5
**10.1-12.4**	76.2	47.7
**10.3-2.4**	76.7	46.3
**10.5-12.4**	76.7	45.8

## 5. Discussion

In the present study, a statistical method, similar to the ROC curve analysis, was adopted to identify a normal range for biomarkers. In this method, some diseases, caused by low or high levels of biomarkers, were considered simultaneously. The significance of this issue would be even more evident when we keep in mind that certain diseases could emerge due to both low and high levels of biomarkers.

Compared to separate analyses obtained by the ROC curve, the present method yields greater sensitivity and specificity levels, particularly when high and Low levels of a biomarker are at stake. Therefore, bearing in mind the pregnancy outcomes of high or low biomarkers, the authors would like to recommend the current method for other biomarkers for which the identification of a normal range is important. Accordingly, identification of a normal range for a biomarker, merely based on clinical observations, without using statistical methods, is not advised.

As low hemoglobin can cause unpleasant discomforts, pregnant women are recommended taking iron supplements to compensate for the deficiency, but this can elevate hemoglobin level and, accordingly, brings about other unpleasant complications. Lower hemoglobin level is causally associated with low birth weight, preterm birth or mortality (11). Furthermore, increase in hemoglobin density during the first half of the pregnancy is a risk factor for contracting preeclampsia (12, 13) and diabetes (14). Therefore, identification of normal range hemoglobin for the first trimester is essential. In this research, the cutoff points were identified as 10 and 12.4, with sensitivity and specificity of 76.2% and 48%, for normal range hemoglobin, during the first trimester.

Most of the previous studies have identified just one cutoff point for hemoglobin (13). A few of them have merely referred to high and low limits, and used clinical experiences instead of statistical methods, to identify a normal range for hemoglobin. In Cunningham’s study, for instance, the normal range of hemoglobin level is between 11 and 12.5 for the first trimester (15). Administration of this range in the current study would yield sensitivity and specificity levels of 73.3% and 45.6%, which are lower than the sensitivity and specificity in this study.

The normal hemoglobin range identified in the present study, has a wider domain, with greater sensitivity and specificity. The point is that because, according to this study, the normal hemoglobin levels are between 10 and 11, then the iron supplements, taken by subjects within this normal range, could lift up their hemoglobin level, subjecting them to pregnancy adverse outcomes. So the normal range for hemoglobin level in Cunningham is not universally practiced in all countries. The mass of evidence supports the practice of routine iron supplementation during pregnancy, although iron supplementation is certainly more important for those pregnant women who have a lower level of hemoglobin (5).

In most studies reviewed here, the classification of hemoglobin was based on clinical experiences (14-16); this indicates that none of the studies carried out earlier used suitable statistical methods to identify a normal range for hemoglobin.

Even when a study had adopted the ROC curve to identify a normal range, the range was limited to one of the high or low level and, therefore, the pregnancy outcomes that are shared to both the high and low values of biomarkers could have unduly affected the identified cutoff points. This indicates that the method in this study can be used to achieve greater sensitivity and specificity in identifying a normal range for the hemoglobin.

In this study, the normal identified range of hemoglobin was based on four pregnancy outcomes and, due to lack of evidence, certain unpleasant results, such as stillbirths and intrauterine growth problems, were not included in the data. The minimum hemoglobin density during the first trimester in this sample was 9 which, of course, those certain unpleasant results, would normally happen in the hemoglobin with very low density (9, 16).

Therefore, further studies, adopting the suggested statistical method, are recommended to consider other unpleasant results due to each of the low levels or high levels of hemoglobin; the data could then be used to identify a normal range of hemoglobin for the first trimester of pregnancy.

In the method, there are no limits for the number of pregnancy outcomes. Thus, in case there is more than one consequence at the same time, the researcher can define a two-state variable in such a way that samples with at least one consequence would be considered sick and be assigned number 1, and samples with no unpleasant consequence would be considered healthy and be assigned number 0.

This article showed many gaps in our knowledge about the normal range of important biomarkers that their normal range was determined by clinical experiences and not by suitable statistical methods. Finally, we suggest using this method for other biomarkers such as hematocrit, blood pressure, FBS, and so on, which their normal ranges have been determined by clinical experiences.
